# Diverse Effects of Natural and Synthetic Surfactants on the Inhibition of *Staphylococcus aureus* Biofilm

**DOI:** 10.3390/pharmaceutics13081172

**Published:** 2021-07-29

**Authors:** Gianna Allegrone, Chiara Ceresa, Maurizio Rinaldi, Letizia Fracchia

**Affiliations:** Department of Pharmaceutical Sciences, Università del Piemonte Orientale “A. Avogadro”, 28100 Novara, Italy; gianna.allegrone@uniupo.it (G.A.); maurizio.rinaldi@uniupo.it (M.R.); letizia.fracchia@uniupo.it (L.F.)

**Keywords:** biosurfactants, rhamnolipids, antimicrobial activity, biofilm dispersal, anti-adhesive/anti-biofilm agents, HPLC-MS

## Abstract

A major challenge in the biomedical field is the creation of materials and coating strategies that effectively limit the onset of biofilm-associated infections on medical devices. Biosurfactants are well known and appreciated for their antimicrobial/anti-adhesive/anti-biofilm properties, low toxicity, and biocompatibility. In this study, the rhamnolipid produced by *Pseudomonas aeruginosa* 89 (R89BS) was characterized by HPLC-MS/MS and its ability to modify cell surface hydrophobicity and membrane permeability as well as its antimicrobial, anti-adhesive, and anti-biofilm activity against *Staphylococcus aureus* were compared to two commonly used surfactants of synthetic origin: Tween^®^ 80 and Triton^TM^ X-100. The R89BS crude extract showed a grade of purity of 91.4% and was composed by 70.6% of mono-rhamnolipids and 20.8% of di-rhamnolipids. The biological activities of R89BS towards *S. aureus* were higher than those of the two synthetic surfactants. In particular, the anti-adhesive and anti-biofilm properties of R89BS and of its purified mono- and di-congeners were similar. R89BS inhibition of *S. aureus* adhesion and biofilm formation was ~97% and 85%, respectively, and resulted in an increased inhibition of about 33% after 6 h and of about 39% after 72 h when compared to their chemical counterparts. These results suggest a possible applicability of R89BS as a protective coating agent to limit implant colonization.

## 1. Introduction

In the past decade, *Staphylococcus aureus* infections have been associated to an increase in hospital stays and mortality and an economic burden estimated at USD 450 million [1,2]. This bacterial species is typically found in the human anterior nasopharynx and skin [3,4] but it is also able to colonize tissue and artificial surfaces (prosthetic orthopedic implants, heart valves, pacemakers, and vascular catheters), causing many acute and chronic persistent infections [5,6]. It can be easily transmitted by direct contact or invasive medical procedures [7,8] and is characterized by a notorious antibiotic recalcitrance, the ability to produce aggressive toxins and virulence factors, and to develop biofilms [9,10]. In its sessile form, *S. aureus* cells live as a structured community embedded in an extracellular polymeric matrix, composed of protein, DNA, and polysaccharide. It can evade host defenses and become tolerant to antimicrobials, making biofilm-associated infections particularly difficult to treat and eradicate [11,12,13]. When associated with medical devices, revision surgery for replacement can be required as well as longer hospital stays, potentially secondary infections, and implant removals may occur [14,15]. Biofilm formation begins with the attachment of bacterial cells to human matrix proteins (collagen, fibronectin, and fibrinogen) covering the devices [12,15]. As consequence, the major challenge in the biomedical field lies in designing new materials and coating strategies to retain bacterial attachment and prevent biofilm formation. This can be obtained principally by modifying the physical properties, such as hydrophobicity/hydrophilicity, texture, charge, and roughness, of abiotic surfaces [10,14,16,17].

Surfactants are the compounds able to lower the interface tension between the substances, and act as detergents, wetting agents, forming agents and dispersants. Surfactants are known to be amphiphilic, due to composing hydrophobic and hydrophilic moiety, and they are classified as cationic, anionic, amphoteric, and non-ionic surfactants [18]. Surfactants have long been used in the cosmetic and food industries as well as in the medical field as cleansing and/or bactericidal solutions [19]. Surfactants can disrupt biofilms themselves and/or perturb the process of their formation [20]. Tween^®^ 80 (polysorbate 80) and Triton^TM^ X-100 are two non-ionic surfactants of synthetic origin, commonly used in laboratories. Polysorbate 80 reduced *Pseudomonas aeruginosa* biofilm formation on medically relevant materials, including contact lenses, at concentrations safe in humans [21]. More recently, a combination of sub-minimal inhibitory concentration (sub-MIC) polymyxin B and polysorbate 80 inhibited biofilm formation of the opportunistic multiple-drug-resistant pathogen *Stenotrophomonas maltophilia* [22]. In addition, an inhibitory effect was demonstrated on plastic, silicone, and dermal tissues on Methicillin-resistant *S. aureus* (MRSA) biofilm formation, suggesting its effectiveness on tissues and foreign bodies as a washing solution for wounds and/or pretreatment of indwelling catheters [20].

Similarly, the nonionic surfactant Triton^TM^ X-100 has been extensively used for its ability to destabilize both eukaryotic and prokaryotic membranes [23,24]. Its toxicity derives from the disrupting action of its polar head group on the hydrogen bonding present within the cell’s lipid bilayer, leading to the destruction of the compactness and integrity of the lipid membrane [25]. Triton^TM^ X-100 has also been demonstrated to induce autolysis by increasing bacteria susceptibility to antibiotics [26,27] and to affect the structure and physiological aspects of biofilms by decreasing the carbohydrate and protein content in the extracellular polymeric substances (EPS) [28].

Among surfactants, biosurfactants (BSs) may represent ideal natural candidates to inhibit the contamination of medical devices by microbial pathogens. In the last decade, a large number of publications has described their anti-adhesive and anti-biofilm properties as coating agents on implantable devices preserving their biocompatibility [29,30,31]. The commercial success of these natural compounds, however, is related to their ability to exhibit equal or superior performance over chemicals. Biosurfactants are characterized by a series of advantages such as low toxicity, high biodegradability, great environmental compatibility, good foaming properties, and stable activity at extreme pH [31,32,33]. Other advantages are the possibility of producing them on renewable media at low cost and of modifying their chemical composition (for example, by means of genetic engineering applied to producing microorganisms) to meet specific functional needs [31,34,35].

These amphiphilic molecules of microbial origin share a pronounced surface activity leading to a reduction of surface tension at the interfaces of phases with dissimilar polarities (liquid–air, liquid–liquid, or liquid–solid) [36,37]. Among them, rhamnolipids are particularly important for applications in the biomedical and technological fields [30,31]. They are mainly produced by *P. aeruginosa* strains and are constituted by a complex mixture of congeners comprising either one (for mono-rhamnolipids) or two (for di-rhamnolipids) molecules of rhamnose glycosidically linked to a dimer of 3-hydroxy fatty acids varying in chain length [38]. The rhamnolipids biosynthetic pathway is distinguished in three major steps [39]. In the first step, the β-hydroxy fatty acids are fused, then two special rhamnosyltransferases sequentially catalyze the condensation of the fatty acid precursors to mono and di rhamnolipids.

The congener distribution has been shown to be mostly strain-dependent but can also be dependent on the growth phase of the culture [39] and on the carbon source used [40].

Rhamnolipids varying in alkyl chain length and rhamnose molecules number have a different hydrophilic-lipophilic balance (HLB) values and therefore may contribute differently on the emulsification properties of the mixture of surfactants.

For the effective evaluation of the rhamnolipids as surfactants, the exact ratio of mono- and di- congeners and the composition in alkyl chains must be known. High-performance liquid chromatography coupled with tandem mass spectrometry (HPLC-MS/MS) is by far the most accurate method for rhamnolipids identification and quantitation [41,42,43,44].

For this reason, in this work, the precise chemical composition and purity of rhamnolipids mixtures produced during the fermentation process has been defined using the HPLC–MS/MS technique.

Then, rhamnolipid R89BS was compared with the chemical surfactants Tween^®^ 80 and Triton^TM^ X-100 to investigate its ability to modify cell surface hydrophobicity and membrane permeability and to evaluate its effectiveness as antimicrobial, anti-adhesive, and anti-biofilm agents against *S. aureus*.

## 2. Materials and Methods

### 2.1. Chemicals and Materials

All solvents and reagents were analytical or the highest grade available. Hydrochloric acid, methanol, acetone, ethanol, and dichloromethane were purchased from Merck (Darmstadt, Germany). Formic and acetic acid, chloroform, and ethyl acetate were purchased from Sigma Aldrich (Milan, Italy). Acetonitrile was purchased from Carlo Erba Chemicals (Rodano, Milan, Italy). Water analytical grade was obtained by ultrafiltration with Elga PureLab Ultra, (M Medical, Cornaredo, Milan, Italy). Triton™ X-100 and Tween^®^ 80 (nonionic surfactants) were purchased from Sigma Aldrich (Milan, Italy).

### 2.2. Bacterial Strains

The biosurfactant-producing strain *P. aeruginosa* 89 was stored at −80 °C in Tryptic Soy Broth (TSB) (Biolife Italiana, Monza, Milan, Italy) with 25% glycerol (Scharlab, Barcelona, Spain). The biofilm producer strain *S. aureus* ATCC^®^ 6538^TM^ was stored at −80 °C in TSB with 25% glycerol.

### 2.3. Biosurfactant Production

Biosurfactant R89 (R89BS) was produced and extracted as described by Ceresa et al. [45]. Briefly, *P. aeruginosa* 89 was grown in Nutrient Broth II (Sifin, Berlin, Germany) at 37 °C for 4 h at 140 rpm. Afterwards, 24 mL of this culture were transferred in 1.2 L of Siegmund-Wagner medium and incubated at 37 °C for 3, 5 and 6 days at 120 rpm. Acidified free-cell supernatant was stored overnight at 4 °C and R89BS extracted three times with ethyl acetate. Then, solvent was evaporated to dryness under vacuum condition and the raw biosurfactant was weighed for yield calculation.

### 2.4. Samples Purification

Rhamnolipid standards used for quantitative analysis and for the biological assays were obtained by purification from the crude extract according to the method reported by Rudden et al. [43] and modified as follows.

The R89BS biosurfactant extract was chromatographed on a silica gel (230–400 mesh; Merck KGa) column. Elution was carried out with dichloromethane and then with dichloromethane/methanol mixture with methanol gradually increased from 5 to 60%. The process was monitored by thin layer chromatography (TLC) that was carried out on precoated silica gel 60 F254 plates (Merck Co., Inc., Damstadt, Germany). TLC plates, spotted with the purified biosurfactant samples dissolved in methanol, were developed using chloroform/methanol/water, 70:28:2 as the mobile phase, and were visualized by spraying plats with non-specific reagent (sulfuric acid/p-anisaldehyde/acetic acid, 1:0.6:50).

The purified fractions contained either only mono-rhamnolipids congeners (fraction I—mR89BS) or only di-rhamnolipids congeners (fraction II—dR89BS). Each fraction was analyzed by LC-MS/MS to determine exact sample composition and each congener was quantified based on the percentage peak area of total rhamnolipids samples (no commercial standards are currently available).

For the LC-MS/MS analysis, an aliquot of the crude biosurfactant extract or of the purified fractions was dissolved in methanol to obtain a 1 mg/mL stock solutions. Working standard solutions were prepared by diluting the stock solutions with methanol to a series of proper concentrations. The standard stock solutions and the working standard solutions were all stored at 4 °C.

### 2.5. LC-MS/MS

A Surveyor HPLC on line with a LCQ DECA XP Plus (Thermo Finnigan, San Jose’, USA) Ion Trap mass spectrometer equipped with an ESI source was employed. For separation, reversed-phase chromatography on an analytical Sinergy Hydro 5 µm C18, 150 × 2 mm column (Phenomenex, Torrance, CA, USA) protected with a C18-Security Guard cartridge, 4 × 2.0 mm (Phenomenex) was used at 30 °C. The flow rate was set to 0.4 mL/min. Mobile phase gradient consisting of 10 mmol/L aqueous ammonium formate buffer at pH 4.2 (A) and acetonitrile (B) was used. The gradient was set to 40% B from 0 to 4 min, increased to 80% B from 4 to 20 min then 90% B from 20 to 26 min, and then it decreased to 40% in 5 min and remained constant for equilibration. The total run time was 36 min. The injection volume was 5 µL.

The mass spectrometer was equipped with an ESI source operating in negative ion mode. The ESI-MS and ESI-MS/MS spectra of the rhamnolipids were recorded using direct infusion of each rhamnolipids solution and the optimal values for ESI source parameters were determined as follows. Source voltage and capillary voltage were at 5.0 kV and −18 V. The capillary temperature was maintained at 300 °C, and nitrogen was used as nebulizing gas at 50 arbitrary units and 7 arbitrary units as auxiliary gas flow. Data were acquired in negative MS total ion scan mode (mass scan range *m/z* 100–1000) and MS/MS product ion scan mode with normalized collision energy (nce%) optimized for each precursor ion selected: *m/z* 475, 531, and 649, 27%, 503, 25%, 677, 29%, and 621, 31%.

LC-ESI-MS/MS in multiple reaction monitoring (MRM) modalities were applied to the selected precursor ions, following the conditions set during the infusion analysis mode. Data were acquired in negative modality.

### 2.6. Rhamnolipids Quantification

Quantitative analyses were performed considering the amount of mono- and di-rhamnolipids congeners present in the purified mR89BS (fractions I) and dR89BS (fraction II), respectively. Each fraction was used as a reference standard: mR89BS as a reference standard of mono-rhamnolipids, dR89BS as a reference standard of di-rhamnolipids.

Calibration curves were measured in triplicate in a range of 1–50 µg/mL (*n* = 5) for each reference fraction (I and II). The limit of detection (LOD) and limit of calculation (LOQ) were determined from the signal-to noise ratios (S/N) with S/N of 3 or greater for LOD and S/N of 10 or greater for LOQ. S/N was calculated for the more abundant congeners of fraction I and II (Rha-C10-C10 and Rha-Rha-C10-C10, respectively).

### 2.7. Determination of Critical Micelle Concentration

Critical micelle concentrations (CMCs) of R89BS extract and purified fraction I and II (mR89BS, dR89BS) were obtained by measuring the surface tension of aqueous biosurfactant solutions from 0.017 to 1 mg/mL. The measurements were carried out at 25 °C with a ring tensiometer (KSV Sigma 703D, KSV Instruments Ltd., Espoo, Finland) according to the Du-Nouy ring method. The value of CMC was defined as the intersection point of the straight lines extrapolated from the curve plotted between biosurfactant concentration and surface tension [46].

### 2.8. Stability Study

The effect of the heat/cold treatments on R89BS solutions (0.5, 1, 2 mg/mL) was evaluated by measuring surface tension and emulsification index at 24 h (E_24_%) after treatment at 100 °C for 1 h and at −80 °C for 24 h. To investigate the pH stability, R89BS solutions at pH ranging from 3.0 to 11.0 were prepared. Afterwards, the surface tension and E_24_% were measured [46].

### 2.9. Antimicrobial Activity (Co-Incubation Assay)

The evaluation of the antibacterial action of synthetic and microbial surfactants against planktonic cells of *S. aureus*^®^ 6538^TM^ was carried out following the protocol of Wiegand et al. [47].

The R89BS stock solution was prepared in phosphate-buffered saline (PBS). The stock solutions of purified fraction I and II (mR89BS, dR89BS) and of synthetic surfactants (Tween^®^ 80, Triton^TM^ X-100) were prepared in PBS at a concentration corresponding to HPLC-MS grade rhamnolipids in R89BS extract. The tested concentrations ranged from 0.03 to 2 mg/mL. *S. aureus* (5 × 10^5^ colony forming unit (CFU)/mL) was grown in Mueller Hinton broth (Sifin, Berlin, Germany) in the presence of surfactants solutions (test wells) or PBS (wells of positive control of growth). Vancomycin (Sigma-Aldrich, Milan, Italy) at 1μg/mL was used as a reference (wells of negative control of growth). Plates were incubated at 37 °C for 16–20 h. Assays were conducted in triplicate and repeated in two independent experiments (*n* = 6).

The MIC of surfactants was defined as the lowest concentration of surfactants that inhibits visible growth of *S. aureus* as observed with the unaided eye.

### 2.10. Cell Surface Hydrophobicity

Bacterial suspension was prepared in PBS to obtain an optical density (OD) at 600 nm of 0.5, treated with the surfactants at their MIC and incubated at 37 °C for 1 h at 150 rpm. Untreated suspension was taken as control. Cell hydrophobicity was measured by microbial adherence to hexadecane (Scharlab, Barcelona, Spain) according to Rosenberg et al. [48]. Microbial cells were collected by centrifugation at 4000 rpm for 15 min and resuspended in PUM buffer, pH 7.1 (22.2 g K_2_HPO_4_·3H_2_O, 7.26 g KH_2_PO_4_, 1.8 g urea, 0.2 g MgSO_4_·7H_2_O and distilled water to 1 L). One milliliter of hexadecane was mixed to 4 mL of cell suspensions in a glass tube at high speed for 2 min and equilibrated for 10 min. Afterward, the OD of the initial cell suspensions and aqueous phases were measured at 550 nm (Genova Plus, Jenway, UK) and cell hydrophobicity was calculated as indicated by Ceresa et al. [49].

Assays were performed in triplicate and repeated in two independent experiments (*n* = 6).

### 2.11. Cell Membrane Permeability

Bacterial suspension was prepared in PBS to obtain an OD at 600 nm of 0.5, treated with the surfactants at their MIC and incubated at 37 °C for 1 h at 150 rpm. Untreated suspension was taken as control. Cell membrane permeability was evaluated by checking crystal violet enhanced penetration. Cells were collected by centrifugation at 4000 rpm for 15 min, resuspended in PBS containing crystal violet (10 µg/mL) and incubated at 37 °C at 150 rpm for 20 min. Cells were collected by centrifugation at 4000 rpm for 15 min and the absorbance of the initial crystal violet (CV) solution and sample solutions was measured at 590 nm (Genova Plus, Jenway, UK). Afterwards, the percentage of crystal violet uptake was calculated as indicated by Ceresa et al. [49].

Assays were performed in triplicate and repeated in two independent experiments (*n* = 6).

### 2.12. Inhibition of Bacterial Adhesion, Biofilm Formation and Disruption of Mature Biofilm on Silicone

#### 2.12.1. Medical-Grade Silicone

Silicone elastomeric discs (SEDs) of two different sizes (10 mm in diameter and 1.5 mm in thickness and 8 mm in diameter and 1.5 mm in thickness) were cut from medical-grade silicone sheets (TECNOEXTR s.r.l, Palazzolo sull’Oglio, Italy) and prepared according to Ceresa et al. [46]. Briefly, SEDs were cleaned with a 1.4% (*v/v*) RBS^TM^ 50 solution (Sigma-Aldrich, Milan, Italy), sonication for 5 min at 60 kHz (Elma S30H, Elmasonic) and two rinses in MilliQ water. Then, SEDs were further sonicated in methanol (99%), rinsed twice, sterilized by autoclaving, and dried at 37 °C for 20 h.

#### 2.12.2. Biofilm Dispersal Activity (Co-Incubation Assay)

Experiments were carried out in 24 well-plates as described by Ceresa et al. [45]. Briefly, SEDs (10 mm in diameter and 1.5 mm in thickness) were moved aseptically into the plates and incubated with 1 mL *S. aureus*^®^ 6538^TM^ culture (1 × 10^7^ CFU/mL in TSB + 1% glucose) at 37 °C in static condition for 24 h. Blank silicone surfaces (without biofilm) were also included in the experimental setting.

The formed biofilms were then incubated in the presence of surfactants solutions (test SEDs) or PBS (control SEDs) for 24 h. The tested surfactants concentrations ranged from 0.06 to 2 mg/mL. At the end of the incubation time, biofilms were washed twice, and their metabolic activity was evaluated by the MTT [3-(4,5-dimethylthiazolyl-2-yl)-2,5-diphenyltetrazolium bromide] reduction assay. Biofilms still present on the surface of SEDs were dipped in 1 mL MTT working solution (0.3% MTT (Sigma-Aldrich), 0,01% glucose, 1 μM menadione (Sigma-Aldrich)) for 30 min at 37 °C. Formazan crystals were dissolved with a solution DMSO/0.1 M glycine buffer (pH 10.2) (7:1). Absorbance at 570 nm (A570) was measured (Victor3VTM, Perkin Elmer, Milan, Italy) and the percentage of biofilm dispersal was calculated. Assays were conducted in triplicate and repeated in two independent experiments (*n* = 6).

#### 2.12.3. Anti-Adhesive Activity (Deposition Assay)

SEDs surface (8 mm in diameter and 1.5 mm in thickness) coating with the BSs was carried out as described by Ceresa et al. [49], with minor changes. Briefly, a volume of 30 μL of surfactants solutions (R89BS: 2 mg/mL; TRITON^TM^ X-100, TWEEN ^®^ 80, mR89BS and dR89BS: at a concentration corresponding to HPLC–MS grade rhamnolipids in R89BS extract) or an equal volume of PBS as control, were deposited on the silicone surfaces. SEDs were then placed under laminar flow to allow complete drying and, subsequently, moved into 48-well plates. The discs were filled with 0.5 mL of a *S. aureus* suspension (10^7^ CFU/mL in TSB +1% glucose) and incubated at 37 °C for 6 h. Blank silicone surfaces (without adherent cells) were also included in the experimental setting.

The quantification of adherent cells was carried out by CV staining. SEDs were filled with 0.5 mL of a 0.2% CV solution for 10 min at room temperature. Afterwards, adherent cells were dissolved with 0.5 mL of 33% acetic acid and A570 was measured. The assays were carried out in triplicate and repeated in two independent experiments (*n* = 6).

#### 2.12.4. Anti-Biofilm Activity (Pre-Coating Assay)

Experiments were carried out as described by Ceresa et al. [45]. Briefly, SEDs (10 mm in diameter and 1.5 mm in thickness) were moved aseptically into 24-well plates. Surfactant-coated SEDs were obtained by immersion in 1 mL of surfactant solutions (R89BS: 2 mg/mL; TRITON^TM^ X-100, TWEEN^®^ 80: at a concentration corresponding to HPLC–MS grade rhamnolipids in R89BS extract) or PBS as control at 37 °C for 24 h at 180 rpm. Subsequently, surfactants solutions or PBS were aspirated, SEDs were moved to new plates and dried under a laminar flow.

Surfactant-coated SEDs and control SEDs were incubated at 37 °C in static conditions with 1 mL *S. aureus*^®^ 6538^TM^ culture (1 × 10^7^ CFU/mL in TBS + 1% glucose). Blank silicone surfaces (without biofilm) were also included in the experimental setting.

Every 24 h, SEDs were transferred onto new plates containing fresh media. The anti-biofilm effect of surfactant-coated SEDs was quantified at different timepoints (24 h, 48 h, 72 h) by CV staining. Biofilms were washed twice, dried and stained with 1 mL of CV solution (0.2%) for 10 min. The dye was solubilized in acetic acid (1 mL, 33%), A570 was measured, and the percentages of biofilm inhibition were calculated.

In addition, the efficacy of purified mono-rhamnolipids congeners (fraction I—mR89BS) and di-rhamnolipids congeners (fraction II—dR89BS) was evaluated at 72 h. BSs-coated SEDs were obtained by immersion in 1 mL of mR89BS and dR89BS solutions (at a concentration corresponding to HPLC–MS grade rhamnolipids in R89BS extract). Biosurfactant-coated SEDs and control SEDs were incubated at 37 °C in static conditions with 1 mL *S. aureus*^®^ 6538^TM^ culture (1 × 10^7^ CFU/mL in TBS + 1% glucose) for 72 h. Every 24 h, SEDs were transferred into new plates containing fresh media. The anti-biofilm effect of surfactant-coated SEDs was quantified by CV staining.

Assays were conducted in triplicate and repeated in two independent experiments (*n* = 6).

### 2.13. Statistical Analysis

All analyses and graphs were performed using the statistical program R, 3.6.2 (R Core Team, 2019). One-way ANOVA, followed by Tukey post hoc test was used to analyse the data obtained in the assays for the evaluation of the activity of surfactants on cell surface hydrophobicity, cell membrane permeability, adhesion, and biofilm formation. Two-way ANOVA, followed by Tukey post hoc test was performed to compare the results obtained from the biofilm dispersal assay and anti-biofilm tests. Results were considered to be statistically significant when *p* < 0.05.

## 3. Results

### 3.1. Chemical Characterization

**Figure 1:**
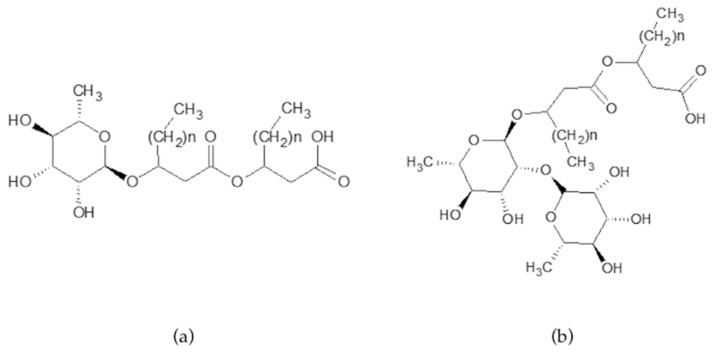
Structure of mono-rhamnolipids (**a**) and di-rhamnolipids (**b**); *n* = 4–8 methylene group for a chain length of C8–C12.

Rhamnolipids are produced as a heterogeneous mixture of mono- and di-rhamnolipids varying in the fatty acid chain length. Mono-rhamnolipids contain one rhamnose moiety whereas di-rhamnolipids contain two rhamnose moieties, connected to two B-hydroxyl acids (Figure 1a,b).

Product ion spectra obtained in tandem mass spectrometry, performed in electrospray ionization (ESI-MS/MS) in negative modality, were used for identification of rhamnolipids and to define the structure of the homologues. The cleavage between the two B-hydroxy fatty acids produce structure informative and complementary fragments.

The mono-rhamnolipid family was composed by five members corresponding of Rha-C10-C10, Rha-C8-C10 and Rha-C10-C8, Rha-C10-C12, and Rha-C12-C10 homologues, whose structures were confirmed by the product ion spectra of the precursor molecules [M–H]^−^ at *m/z* 503, 475, and 531, respectively.

**Figure 2:**
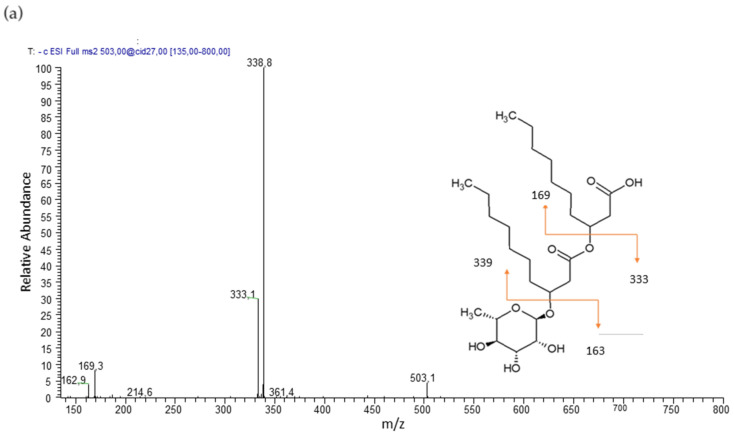

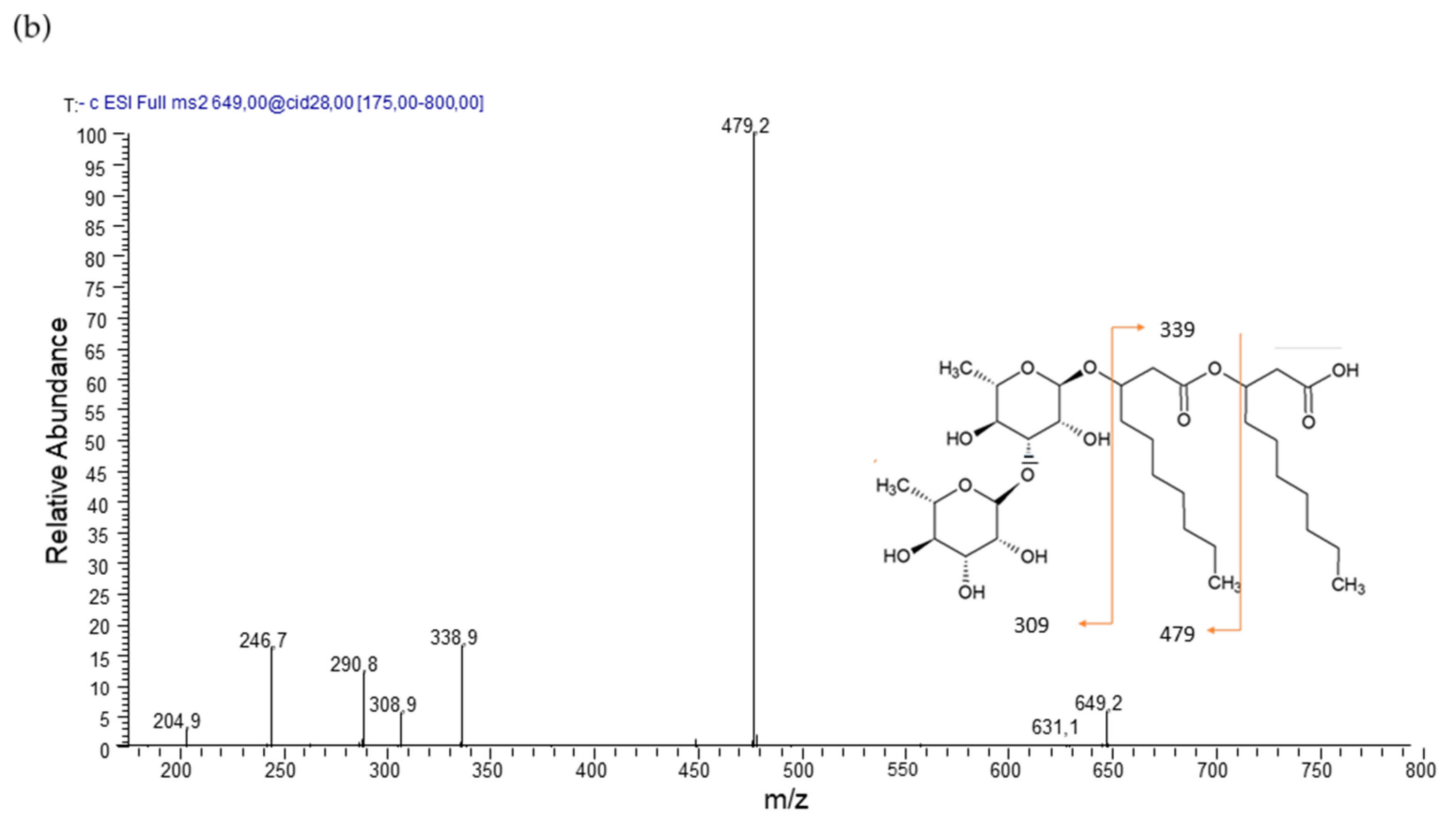
Product ion spectra and proposed fragmentation of (**a**) *m/z* 503 [M–H]^−^ of Rha-C10-C10 and (**b**) *m/z* 649 [M–H]^−^ of Rha-Rha-C10-C10.

Mono-rhamnolipid Rha-C10-C10, precursor molecules [M–H]^−^ at *m/z* 503 shows a product ion spectrum with fragment [Rha-C10]^−^ (*m/z* 333) after cleavage of the terminal B-hydroxy fatty acid. Further fragmentation is [C10-C10]^−^ (*m/z* 339) (Figure 2a). Mono-rhamnolipids Rha-C10-C8 and Rha-C8-C10, precursor molecule [M–H]^−^ at *m/z* 475 show a product ion spectrum with fragment [Rha-C10]^−^ (*m/z* 333) or [Rha-C8]^−^ (*m/z* 305) after cleavage of the terminal B-hydroxy fatty acid. Further fragmentation is [C10-C8]^−^/[C8-C10]^−^ (*m/z* 311). Mono-rhamnolipids Rha-C12-C10 and Rha-C10-C12, precursor molecule [M–H]^−^ at *m/z* 531 show a product ion spectrum with fragment [Rha-C12]^−^ (*m/z* 361) or [Rha-C10]^−^ (*m/z* 333) after cleavage of the terminal B-hydroxy fatty acid. Further fragmentation is [C12-C10]^−^/[C10-C12]^−^ (*m/z* 367).

The di-rhamnolipid family was represented by three main members corresponding Rha-Rha-C10-C10, Rha-Rha-C10-C12, and Rha-Rha-C12-C10 homologues, whose structures were confirmed by the product ion spectra of the deprotonated molecules [M–H]^−^ at *m/z* 649 and 677, respectively.

Di-rhamnolipid Rha-Rha-C10-C10, precursor molecule [M–H]^−^ at *m/z* 649 shows a product ion spectrum with fragment [Rha-Rha-C10]^−^ (*m/z* 479) after cleavage of the terminal B-hydroxy fatty acid. Further fragmentation is [C10-C10]^−^ (*m/z* 339) (Figure 2b). Di-rhamnolipids Rha-Rha-C12-C10 and Rha-Rha-C10-C12, precursor molecule [M–H]^−^ at *m/z* 677 shows a product ion spectrum with fragment [Rha-Rha-C12]^−^ (*m/z* 507) or [Rha-Rha-C10]^−^ (*m/z* 479) after cleavage of the terminal B-hydroxy fatty acid. Further fragmentation is [C12-C10]^−^/[C10-C12]^−^ (*m/z* 367).

LC-ESI-MS/MS analysis was set up to analyse purified fraction I (mR89BS), purified fraction II (dR89BS), and R89BS crude extracts. Table 1 summarises the MRMs experimental conditions obtained during the set up and subsequently used for the quantification studies. In mR89BS the deprotonated ion at *m/z* 503 corresponds to Rha-C10-C10, the most abundant congener (90.1%) of the total of the sample. In dR89BS the deprotonated ion at *m/z* 649 corresponds to Rha-Rha-C10-C10, the most abundant congener (96.8%) of the total of the sample.

**Figure 3:**
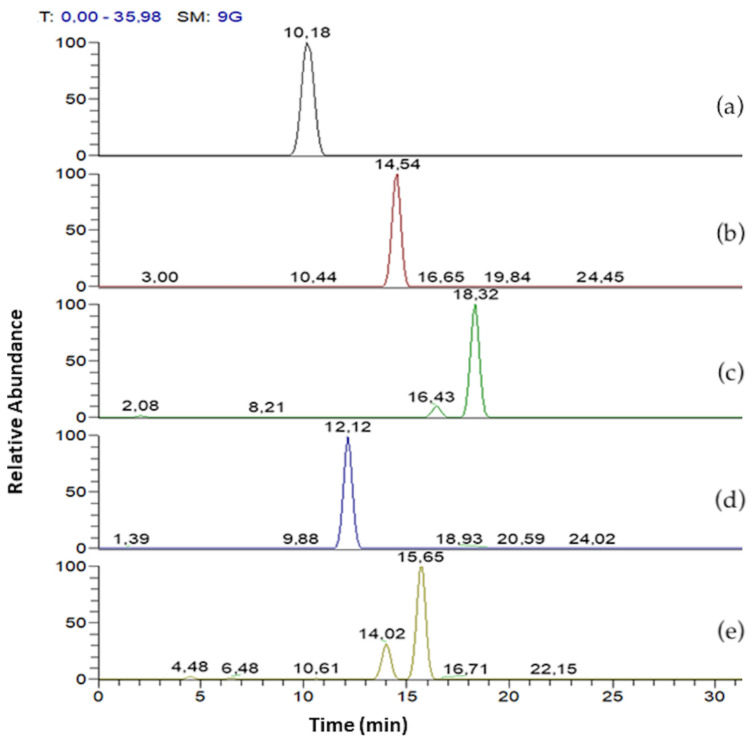
LC-MS/MS mass chromatogram of R89BS crude extract. Mono-rhamnolipids with [M–H]^−^ at *m/z* 475 (**a**), 503 (**b**), 531 (**c**). Di-rhamnolipids with [M–H]^−^ at *m/z* 649 (**d**) and 677 (**e**).

In Figure 3 mono- and di-rhamnolipids congeners of R89BS crude extract are separated according to the following Rt as reported in Table 1. Mono-rhamnolipids Rt were 10.18, 14.54, 18.32 min and di rhamnolipids Rt were 12.12 and 15.65 min, respectively. Differentiation on constitutional isomers (Rha-C10-C8 and Rha-C8-C10, Rha-C10-C12 and Rha-C12-C10, Rha-Rha-C10-C12 and Rha-Rha-C12-C10) were not possible to separate using chromatography but can be achieved by means of MS/MS experiment.

### 3.2. Rhamnolipids Quantification

Purified fraction I (mR89BS) and purified fraction II (dR89BS) were used as reference standard of mono-rhamnolipid and di-rhamnolipids quantification, respectively. Mono-rhamnolipids fraction, represented by congener with pseudo molecular ions [M-H]^−^ 475, 503, 531 *m/z* was used for the construction of mono-rhamnolipids calibration curve injecting five different concentration levels and analysing in triplicate. It showed a good linearity in the range 1–50 µg/mL. LOD and LOQ were evaluated for the more abundant congener of the fraction, Rha-C10-C10, and were found to be 0.05 and 0.1 µg/mL.

Di-rhamnolipids fraction, represented by congener with pseudo-molecular ions [M-H]^−^ 649, 677 *m/z* was used for the construction of di-rhamnolipids calibration curve injecting five different concentration levels and analysing in triplicate. It showed a good linearity in the range 1–50 µg/mL. LOD and LOQ were evaluated for the more abundant congener of the fraction, Rha-Rha-C10-C10, and were found to be 0.05 and 0.1 µg/mL.

The method was applied for the quantitation and comparison of rhamnolipids congeners present in R89BS crude extracts obtained at different incubation times. Table 2 shows the concentrations of rhamnolipids congeners obtained after 3-, 5-, and 6-days incubation time. The higher amount of rhamnolipids congeners produced (91.4% of the entire extract) was obtained after 5 days incubation time, against 55.8% after 3 days and 76.8% after 6 days. In all extracts, Rha-C10-C10 (MW 504 Da) and Rha-Rha-C10-C10 (650 Da) are the most abundant congeners, while the mono-rhamnolipids family represents the more abundant family (varying from 59.7 to 77.2%).

The grade of purity of the R89BS crude extract obtained with 5 days incubation was 91.4% and was composed by 70.6% of mono-rhamnolipids and 20.8% of di-rhamnolipids. R89BS crude extract and its purified mR89BS and dR89BS were tested for anti-adhesive and anti-biofilm assays.

### 3.3. Determination of Critical Micelle Concentration and Stability Study

At pH 7.0, the condition adopted in anti-biofilm assays, the experimental results revealed that the surface tension of alkaline distilled water decreased rapidly as the concentration of biosurfactant increased with a minimum surface tension of 29.65 mN/m, 29.32 mN/m, and 34.76 mN/m for the R89BS crude extract, purified fraction I (mR89BS), and purified fraction II (dR89BS), respectively.

CMC values for R89BS, mR89BS, and dR89BS were found at 0.0563 mM, 0.0606 mM, and 0.0461 mM, respectively.

In addition, it was observed that R89BS solutions retained their surface activity over a wide pH range and heat treatments. In particular, at the pH range from 3.0 to 11.0, the surface tension is preserved without significant variations with an average value of 30.64 ± 0.30 mN/m. On the contrary, E_24h_% is not altered to pH values between 7.0 and 11.0, showing just over 60%, but it is zero from pH 6.0 at pH 3.0 (Table S1). The treatments at 100 °C for 1 h and at −80 °C for 24 h did not cause any particular changes in surface tension and emulsion index compared to the values obtained with R89BS solutions maintained at room temperature (20 °C) and used as a control.

### 3.4. Antimicrobial Activity

To evaluate and compare the antimicrobial activity of R89BS, mR89BS, dR89BS, Triton^TM^ X-100 and Tween^®^ 80, bacterial planktonic cells were treated with different concentrations of the five surfactants for 16–20 h. An antibacterial activity against *S. aureus*^®^ 6538^TM^ planktonic cells was found for R89BS, mR89BS and Triton^TM^ X-100. MIC values for these surfactants were identified in the concentration of 0.06 mg/mL, 0.03 mg/mL, and 0.22 mg/mL respectively. On the contrary, no MIC was detected for dR89BS and Tween^®^ 80.

### 3.5. Cell Surface Hydrophobicity and Cell Membrane Permeability

**Figure 4:**
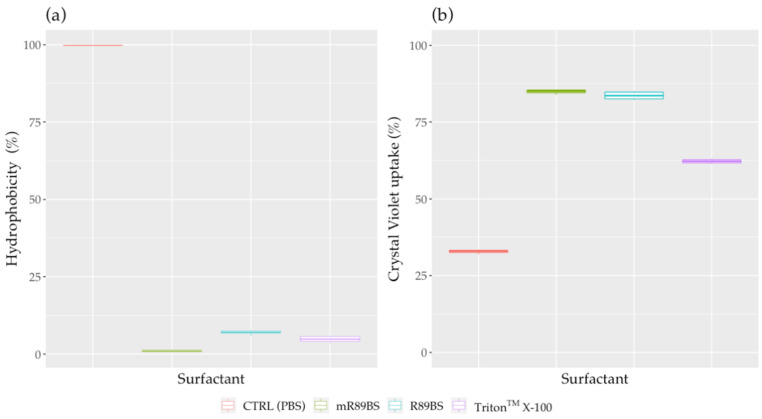
Changes in surface hydrophobicity and crystal violet uptake induced by surfactants. Surface hydrophobicity (**a**) and crystal violet uptake (**b**) of *S. aureus*^®^ 6538^TM^ cells treated with natural (R89BS, mR89BS) and chemical surfactants (Triton^TM^ X-100), compared to control samples.

Cell surface hydrophobicity and cell membrane permeabilization studies were performed by treating *S. aureus*^®^ 6538^TM^ with surfactants at their MIC: for this reason, dR89BS and Tween^®^ 80 were excluded from these assays. As shown in Figure 4a, there were significant differences in *S. aureus*^®^ 6538^TM^ surface hydrophobicity (CSH) between control samples and bacterial cells incubated with R89BS, mR89BS, and Triton^TM^ X-100 MIC concentrations (*p* < 0.001). In particular, the treatment with these three surfactants induced a decrease of CSH from 99.8% (control samples) to 1% for mR89BS, 7% for R89BS, and 4.8% for Triton^TM^ X-100. Furthermore, significant changes in *S. aureus* cell membrane permeability were also observed for the three surfactants (*p* < 0.001) with an increase in crystal violet uptake from 32.8% (control samples) to 62.2% for Triton^TM^ X-100, 83.6% for R89BS and 84.9% for mR89BS (Figure 4b).

### 3.6. Biofilm Dispersal

**Figure 5:**
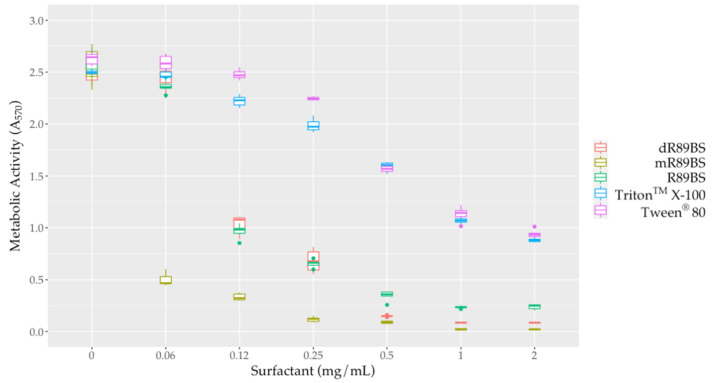
Metabolic activity of *S. aureus*^®^ 6538^TM^ biofilms after treatment with surfactants. To evaluate the dislodging effect of surfactants, pre-formed biofilms were co-incubated with increasing concentrations of the different samples for 24 h at 37 °C.

To evaluate the effect of surfactants against sessile cells, biofilms were co-incubated for 24 h with different concentrations of R89BS, mR89BS, dR89BS, Triton^TM^ X-100 and Tween^®^ 80. All the tested surfactants were able to dislodge *S. aureus*^®^ 6538^TM^ pre-formed biofilms (Figure 5). R89BS, dR89BS, Triton^TM^ X-100 and Tween^®^ 80 are ineffective at the concentration of 0.06 mg/mL but promoted biofilm dispersal at concentrations from 0.12 to 2 mg/mL with a reduction of the biofilm metabolic activity in a range of 68–89% for R89BS, 58–97% for dR89BS, and 6–65% for Triton^TM^ X-100 and Tween^®^ 80. mR89BS, instead, induced biofilm dispersal at all the tested concentrations with reductions ranging from 80% to 99%. As confirmed by two-way ANOVA, followed by Tukey post hoc test, the result was significantly dependent on the type of surfactant and on the tested concentration (*p* < 0.001). R89BS and its purified fractions were the most effective: the values of absorbance derived from the biofilms treated with the biosurfactants at concentrations ranging from 0.12 to 2 mg/mL were significantly different to those obtained with the chemical surfactants (*p* < 0.001). Triton^TM^ X-100 and Tween^®^ 80 showed a similar dislodging activity (*p* > 0.05).

### 3.7. Anti-Adhesive Activity

**Figure 6:**
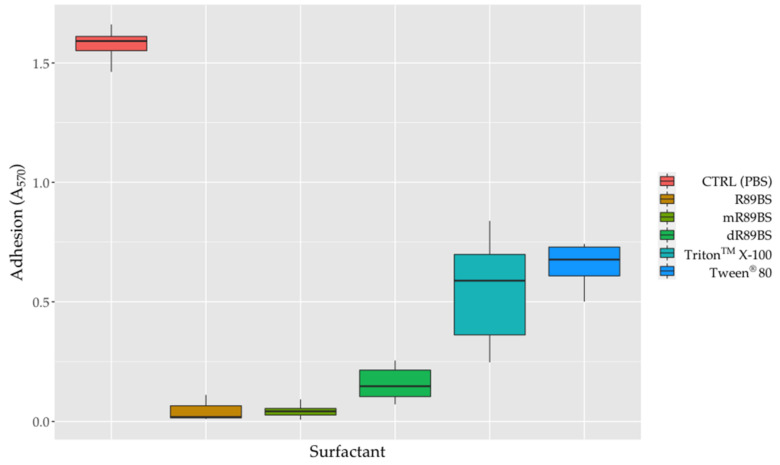
Bacterial adhesion on silicone discs. The efficacy of the surfactants surface treatment on SEDs was evaluated as reduction of *S. aureus*^®^ 6538^TM^ adherent cells after 6h of incubation at 37 °C, compared to controls SEDs.

To evaluate the anti-adhesive properties of surfactants, SEDs were coated with the R89BS crude extract, the purified fractions I and II (mR89BS, dR89BS) and the two chemical surfactants. As confirmed by one-way ANOVA, bacterial adhesion on SEDs was significantly dependent on the treatment of surfactants (*p* < 0.001). In general, surfactant-coatings efficiently limit the adhesion of *S. aureus*^®^ 6538^TM^ on silicone surfaces (Figure 6). As shown, the bacterial cells that adhere on all surfactant-coated SEDs were lower to those that were present on the controls. After 6 h, with respect to uncoated control SEDs, the pre-treatment of silicone surfaces with R89BS, mR89BS, dR89BS, Triton^TM^ X-100 and Tween^®^ 80 resulted in a reduction in cell adhesion of 97%, 97%, 90%, 65% and 58%, respectively. In particular, as confirmed by Tukey post hoc test, R89BS and its purified fractions showed absorbance values significantly different to those observed with the chemical surfactants (*p* < 0.001). Conversely, no differences were found among R89BS, mR89BS and dR89BS as well as between Triton^TM^ X-100 and Tween^®^ 80 (*p* > 0.05).

### 3.8. Anti-Biofilm Activity

**Figure 7:**
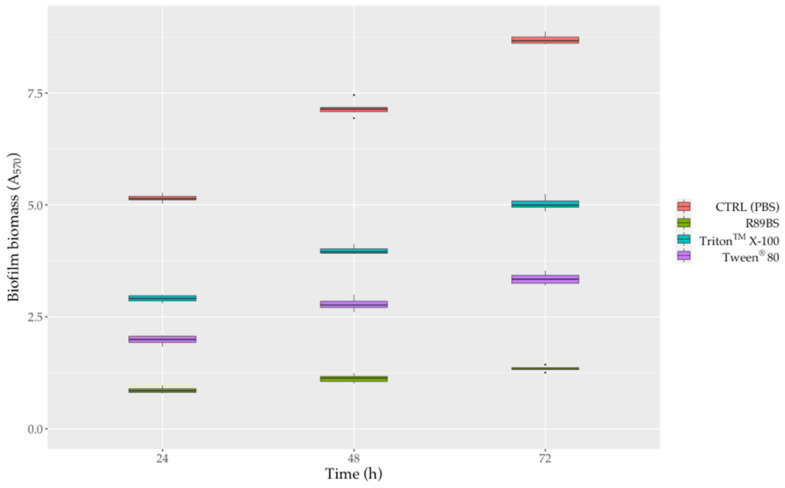
Activity of surfactant-coated SEDs against *S. aureus*^®^ 6538^TM^ biofilm formation. The anti-biofilm efficacy of surfactant-coated SEDs was evaluated in terms of inhibition of the total biofilm biomass after 24, 48 and 72 h of incubation.

In general, surfactant-coated SEDs also efficiently counteract *S. aureus*^®^ 6538^TM^ biofilm formation up to 72 h. The anti-biofilm activity of surfactant-coated SEDs against *S. aureus* biofilm growth was shown in Figure 7 and Figure S1. After 72 h, with respect to uncoated control SEDs, the pre-treatment of silicone surfaces with R89BS, Tween^®^ 80 and Triton^TM^ X-100 resulted in a biofilm biomass reduction of 84.6%, 61.5% and 42.3%, respectively (Figure 7). However, as confirmed by two-way ANOVA followed by the Tukey post-hoc test, the differences in biofilm development on SEDs was significantly dependent on the type of surfactant used for the pre-treatment of the silicone surfaces (*p* < 0.001). In particular, it was observed that R89BS-coated discs were the most effective in inhibiting *S. aureus* biofilm growth, followed by Tween^®^ 80-coated and Triton^TM^ X-100-coated discs. Conversely, no differences were found among R89BS, mR89BS, and dR89BS (*p* > 0.05). In addition, it was interesting to note that the biofilm inhibitory activity of all the surfactants tested (both synthetic and natural) remained almost constant over time (from 24 to 72 h).

## 4. Discussion

In this study, an accurate and specific HPLC-MS/MS method has been applied for the identification and quantitation of rhamnolipids congeners produced by *P. aeruginosa*. R89BS crude extract (purity 91.4%) was composed by five mono-rhamnolipids (70.6%) and three di-rhamnolipids (20.8%) congeners, ranging from C8 to C12 chain length, with congeners Rha-C10-C10 and Rha-Rha-C10-C10 as the most abundant.

The composition of R89BS crude extract studied at different incubation times shows that the mono rhamnolipid family was always the most prevalent. In particular, the highest concentration was observed at five days of incubation, corresponding to the transition to the stationary phase and reflecting their secondary metabolite behavior. On the contrary, di-rhamnolipids were almost constant at the three time points. The molar ratio of mono- and di-rhamnolipids is known to be strain dependent and has been demonstrated to vary over time [50].

R89BS and its purified fractions showed a high reduction of water surface tension and low critical micelle concentration. The CMC of rhamnolipids is quite low if compared to other biosurfactants; however, differences in their surface tension and CMC were reported with values, respectively, in the range of 72–30 mN/m and of 10.1–500 mg/L [51].

The hydrophilic/lipophilic balance (HLB) values were calculated according to Griffin’s method for non-ionic surfactants [52]. HLB were 6.5 and 9.5 for Rha-C10-C10 and Rha-Rha-C10-C10, respectively, as also observed by Diaz De Rienzo et al. [52]. The analysis of the rhamnolipids produced by *Burkholderia thailandensis* E264 (Rha-Rha-C14 -C14) and *P. aeruginosa* ATCC 9027 (Rha-C10 -C10) had an HLB values of 9.2 and 6.5, respectively [53]. Therefore, R89BS mixture shows an HLB value of 7.2 calculated as a weighted average by considering the percentage composition of the extract. However, under the condition tested in this work (pH 7.0), R89BS carboxylic moieties are dissociated. In this case, due to rhamnolipid ionization, the real HLB value may be higher, increasing its emulsification activity as confirmed by the E_24_% index, which was of about 60% in the pH range 7–11 and zero at lower pH (Table S1).

The antibacterial activity of R89BS, mR89BS, and dR89BS was evaluated in co-incubation condition and compared with that of commonly used synthetic surfactants, such as Tween^®^ 80 and Triton^TM^ X-100 [54,55].

A minimal inhibitory concentration was detected only for R89BS, mR89BS, and Triton^TM^ X-100 but not for Tween^®^ 80 and dR89BS. From this experimental evidence, it is possible to assume that the antibacterial activity of the crude extract is ascribed to the mono-rhamnolipid fraction. In particular, R89BS and mR89BS were more efficient in killing *S. aureus* planktonic cells compared to Triton^TM^ X-100, as confirmed by their higher cell permeabilization ability. These results suggest that the inhibition subsequently observed on *S. aureus* biofilm formation may also depend on the ability of these three surfactants to affect the pathogen growth.

The antimicrobial activity of rhamnolipids and other types of glycolipids was previously reported [56,57,58,59]. The glycolipid obtained by the marine strain *Staphylococcus saprophyticus* SBPS 15 completely inhibited the growth of human pathogens such as *Escherichia coli*, *Klepsiella pneumoniae, P. aeruginosa*, *Vibrio cholerae*, *S. aureus*, *Candida albicans* at concentrations of 4–64 μg/mL [60]. Sophorolipids (SLs)-grafted gold monolayers showed biocidal activity against both Gram-positive (*Enterococcus faecalis*, *Staphylococcus epidermidis*, *Streptococcus pyogenes*) and Gram-negative (*E. coli*, *P. aeruginosa* and *Salmonella typhymurium*) strains [61].

In addition, R89BS, mR89BS, and Triton^TM^ X-100 were able to strongly decrease *S. aureus* cell surface hydrophobicity, which may affect cells ability to adhere to surfaces. It is known that hydrophobicity of cells can increase the propensity of microorganisms to adhesion, causing damage to surfaces by biofilm formation, particularly to medical implants that are constructed from hydrophobic materials such as silicone, stainless steel, Teflon, etc. [62].

After comparative analysis of commercial rhamnolipids activity on resistant and sensitive bacteria, de Freitas Ferreira et al. [63] suggested that these biosurfactants promotes alterations in proteins, carbohydrates, and membrane phospholipids present on the cell surface by solubilization and release of apolar membrane components or adsorption of rhamnolipids on cell surface. These alterations probably cause the observed reduction in cell surface hydrophobicity, the changes in cell permeability or cell lysis, as confirmed by scanning electron microscopy.

On the contrary, for Tween^®^ 80 and dR89BS, no minimal inhibitory concentration was found on planktonic cells in the tested concentration range, suggesting that the inhibition of biofilm formation as well as the biofilm disintegrating activity observed subsequently do not depend on its ability to strongly influence the pathogen growth.

All the surfactants showed significant activity against *S. aureus* sessile cells in co-incubation experiments, where R89BS was the most efficient. This activity is also defined as the ability to disrupt pre-formed biofilm [64,65,66] and it seems to be due to the direct antimicrobial action of biosurfactants against pathogens but also to cavity formation within the biofilm structures [67] and/or to the induction of changes in the quorum sensing signaling and gene expression involved in biofilm growth and dispersal [68,69,70].

Liu et al. [71] demonstrated that surfactin affected *S. aureus* adhesion and significantly promoted biofilm dislodging, decreasing the production of alkali-soluble polysaccharides, the downregulation of icaA and icaD expression, and the alteration of the quorum sensing system by the regulation of the auto inducer 2 activity. The effect of different types of rhamnolipids and sophorolipids was also investigated against some oral bacterial pathogens such as *Streptococcus oralis, Actinomyces naeslundii, Neisseria mucosa* and *Streptococcus sanguinis* [72,73]. These glycolipids significantly inhibited the formation of biofilms in a range of 60–90% and dislodge pre-existing biofilms in a range of 50–100%.

The anti-adhesive and anti-biofilm activities of R89BS were evaluated on silicone at different incubation times and compared to those of Triton^TM^ X-100 and Tween^®^ 80 synthetic surfactants. Purified fractions containing only mono- (Rha-C10-C10 90.1%) or di-rhamnolipids (Rha-Rha-C10-C10 96.9%) were also prepared to test their activity in comparison with the crude extract and to detect which of the two fractions was the most efficient.

R89BS crude extract showed the highest ability to inhibit *S. aureus* adhesion to silicone and its activity was not significantly different from that of the mono- and di-rhamnolipid purified fractions, suggesting that they contribute equally to the anti-adhesive activity of the biosurfactant. Similarly, the biosurfactant R89BS showed a better ability to inhibit the formation of *S. aureus*^®^ ATCC 6538^TM^ biofilm compared to the synthetic surfactants at all incubation times. Moreover, its efficacy was also confirmed by the fact that the real amount of R89BS adhered to the silicone surface was only 4.4 μg for disc, as measured through the HPLC-MS/MS method after washing the coated discs. R89BS coating on silicone, as confirmed by the previously observed reduction of the advancing and receding contact angle [45], significantly increased the surface wettability and hydrophilicity preventing microbial adhesion. As in this study, other research groups demonstrated that biosurfactants are able to alter the chemical and physical properties of surfaces such as their roughness and hydrophobicity, counteracting microbial adhesion and biofilm formation [74,75].

Triton^TM^ X-100 and Tween^®^ 80 anti-adhesive activity was almost similar, whereas Triton^TM^ X-100 had the mildest action at inhibiting the formation of *S. aureus* biofilm. Furthermore, the biofilm inhibitory activity of all the surfactants tested (both synthetic and natural) remained almost constant over time (from 24 to 72 h).

Even if the anti-biofilm activity of glycolipids is well known [72,76,77,78], in this work, for the first time, the higher anti-biofilm efficiency of rhamnolipids compared to synthetic surfactants was demonstrated. Similar results were reported by Pontes et al. [79] on other glycolipids. These authors compared the ability of a mixture of sophorolipids, produced by *Starmerella bombicola* CBS 6009, to prevent biofilm formation of *S. aureus* and *E. coli* to the synthetic surfactants Triton^TM^ X-100 and sodium dodecyl sulfate (SDS). Both synthetic surfactants showed lower anti-biofilm activity than that of the biosurfactant at 24 h of incubation. In addition, similarly to R89BS sophorolipids decreased the hydrophobicity of the silicone surface, thus limiting biofilm formation of *S. aureus* and *E. coli* [79].

Further evidence of the efficiency of R89BS as a coating agent was reported by Tambone et al. [80]. The rhamnolipid coating significantly inhibited *Staphylococcus spp.* biofilm formation on three commercial titanium implant surfaces, leading to a decrease in terms of biomass up to 98% for *S. aureus*, and up to 78% for *S. epidermidis* at 24 h.

R89BS also showed anti-adhesive and anti-biofilm properties against inter-kingdom dual-species biofilms (*S. aureus*-*C. albicans* and *S. epidermidis*-*C. albicans*), inducing a remarkable reduction of biofilm biomass, metabolic activity, viability, and microstructural architecture, up to 72 h on both polystyrene and silicone surfaces without affecting the biocompatibility of the material [49].

## 5. Conclusions

In the present study, the rhamnolipid produced by *P. aeruginosa* R89 was deeply characterized and its antimicrobial/anti-biofilm activities compared with commercial synthetic surfactants, namely Triton X-100 and Tween 80.

Rhamnolipid R89BS, composed of a mixture of mono-rhamnolipids (70.6%) and di-rhamnolipids (20.8%), showed a high water surface tension reduction, a low critical micelle concentration, and good emulsification activity, thermal resistance, and pH stability. R89BS and its purified fraction mR89BS demonstrated antibacterial activity with low MIC values (0.06 µg/mL and 0.03 µg/mL, respectively).

R89BS mixture inhibited *S. aureus* adhesion and biofilm formation on medical-grade silicone more effectively than the synthetic surfactants, up to 72 h incubation.

R89BS mixture and the purified mR89BS were able to alter the *S. aureus* membrane permeability more efficiently than Triton X^TM^-100 and to modify the surface properties of both cells and silicone, decreasing their hydrophobicity, and therefore the adhesive properties. Both mono- and di-rhamnolipid fractions showed the same activity of the crude extract on the *S. aureus* adhesion, suggesting that time-consuming and expensive purification and separation procedures are not necessary.

The results obtained, together with the previous observations of biocompatibility and activity against mono- and dual-species biofilms on silicone and titanium, further endorse the idea of a possible applicability of these natural molecules as a coating agent to limit microbial adhesion and the onset of biofilm. In summary, the use of R89BS may be envisaged as a protective measure and support to antimicrobial therapy, to reduce implant colonization and mitigate infections related to biomaterials for medical use.

## Figures and Tables

**Table 1:** LC-MS/MS MRM parameters for rhamnolipids congeners analysis and relative abundance in purified fractions.

Rhamnolipids Congeners	Rt min	[M-H]^−^ *m/z*	MS/MS (nce% ^1^) *m/z*	Abundance %
mR89BS (fraction I)				
Rha - C10 - C8/C8 - C10	10.18	475	305, 333, 311 (27)	2.8
Rha - C10 - C10	14.54	503	333, 339 (27)	90.1
Rha - C12 - C10/C10 - C12	18.32	531	333, 361, 367 (27)	7.1
Total				100
dR89BS (fraction II)				
Rha - Rha - C10 - C10	12.12	649	339, 479 (28)	96.9
Rha - Rha - C10 - C12/C12 - C10	15.65	677	507, 479, 367 (29)	3.1
Total				100

^1^ nce%: normalized collision energy.

**Table 2:** Concentrations of rhamnolipids congeners in R89BS crude extracts at different incubation times (3, 5 and 6 days).

Rhamnolipids Congeners	3 Days mg/g (sd ^1^)	5 Days mg/g (sd)	6 Days mg/g (sd)
Mono-rhamnolipids			
Rha-C10-C8/C8-C10	9.22 (0.93)	19.53 (1.93)	14.93 (1.34)
Rha-C10-C10	299.93 (15.12)	635.88 (33.70)	485.48 (23.64)
Rha-C12-C10/C10-C12	2.84 (0.25)	50.55 (5.35)	38.59 (3.72)
Total	311.99	705.96	539.00
Di-rhamnolipids			
Rha-Rha-C10-C10	217.80 (17.21)	201.67 (16.34)	205.20 (16.22)
Rha-Rha-C10-C12/C12-C10	7.21 (0.71)	6.67 (0.88)	24.13 (2.97)
Total	225.01	208.34	229.33
			
Total rhamnolipids	537.00	914.30	768.33

^1^ sd: standard deviation.

## Data Availability

All data presented in this study are included in the submitted manuscript.

## Supplementary Materials

The following are available online at https://www.mdpi.com/article/10.3390/pharmaceutics13081172/s1, Additional information on R89BS chemical characterization. Table S1: Effect of pH on R89BS surface tension and E_24h_%. Figure S1: Activity of purified mono-/di-rhamnolipids-coated SEDs against *S. aureus*^®^ 6538^TM^ biofilm formation.
