# Supplementary material for: Diverse Effects of Natural and Synthetic Surfactants on the Inhibition of Staphylococcus aureus Biofilm
Source: Pharmaceutics. 2021 Jul 29;13(8):1172. doi: 10.3390/pharmaceutics13081172 (PMC8402037; doi:10.3390/pharmaceutics13081172)
Supplement: Supplementary file 1 [file pharmaceutics-13-01172-s001.zip › pharmaceutics-1289274-supplementary.pdf]

pharmaceutics

Supplementary Materials: Diverse Effects of Natural and Synthetic Surfactants on the Inhibition of *Staphylococcus aureus* Biofilm

Gianna Allegrone, Chiara Ceresa, Maurizio Rinaldi and Letizia Fracchia

3.1. Chemical Characterization

During development of a suitable LC-ESI-MS/MS method for qualitative and quantitative determination of the rhamnolipids in R89BS extract, preliminary tests were performed with the objective of selecting the adequate and optimum conditions. Among the parameters to be optimized, the stationary phase of the used column and the choice of aqueous and organic eluent are the most important. The organic eluent was varied between acetonitrile and methanol with best results obtained for acetonitrile. The aqueous eluent was chosen to be acidic and best results were obtained with ammonium formate buffer. Sinergy Hydro RP column gives the best results as stationary phase to separate rhamnolipid congeners.

Mass spectrometry detection, performed in electrospray ionization (ESI) negative mode, since carboxylic groups are suitable to form pseudo molecular ions $[M - H]^-$, was first devoted to obtain MS-based information for the simultaneous evaluation of rhamnolipids. Secondly, to define the structure of the homologues and to optimize the MRM assay, the ESI-MS/MS spectra were performed.

3.3. Determination of Critical Micelle Concentration and Stability Study

Table S1. Effect of pH on R89BS surface tension and E_{24h}%.

**pH**	**0.5 mg/mL**			**1 mg/mL**			**2 mg/mL**		
	**Surface tension (mN/m)**		E_{24h} %	**Surface tension (mN/m)**		E_{24h} %	**Surface tension (mN/m)**		E_{24h} %
	Mean	sd[1]		Mean	sd		Mean	sd	
3.0	29.29	0.88	0	29.90	0.07	0	29.61	0.28	0
4.0	29.41	0.31	0	29.54	0.19	0	30.41	0.43	0
5.0	29.74	0.31	0	29.62	0.58	0	29.52	0.46	0
6.0	29.74	0.47	0	29.47	0.11	0	29.21	0.16	0
7.0	30.90	0.25	61	31.20	0.11	61	31.17	0.14	61
8.0	31.80	0.27	62	31.00	0.60	62	31.13	0.18	62
9.0	31.67	0.19	62	31.32	0.24	62	31.25	0.23	62
10.0	31.66	0.35	62	31.57	0.35	63	31.66	0.27	63
11.0	32.16	0.24	63	32.20	0.11	63	31.68	0.29	63

[1] sd: standard deviation.

3.8. Anti-Biofilm Activity

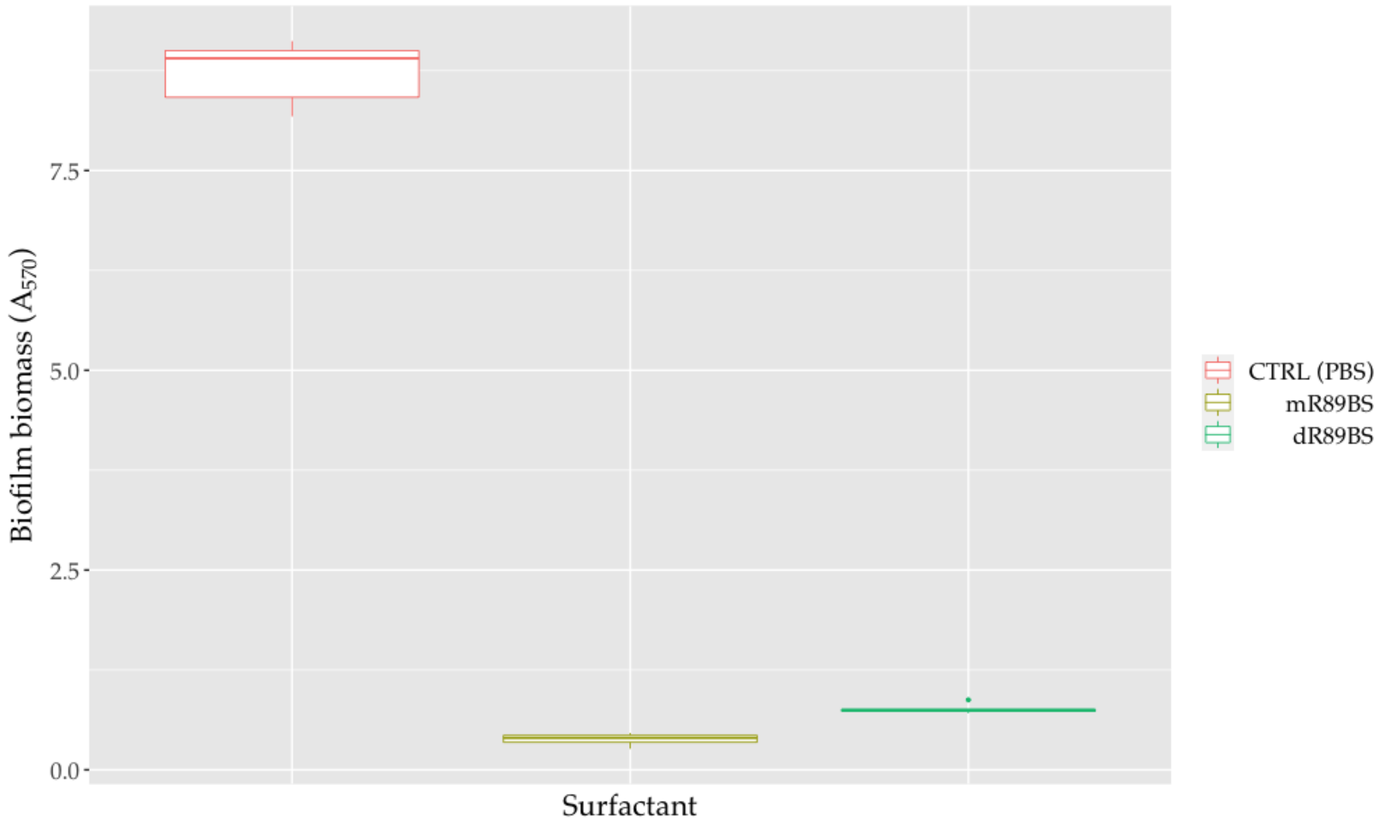

Figure S1. Activity of purified mono-/di-rhamnolipids-coated SEDs against *S. aureus*® 6538™ biofilm formation. The silicone surfaces were coated with an mR89BS or dR89BS solution (at a concentration corresponding to HPLC/MS-grade rhamnolipids in R89BS extract) by means of direct physical adsorption. The anti-biofilm efficacy of mR89BS/dR89BS-coated SEDs, compared to control SEDs, was evaluated in terms of inhibition of the total biofilm biomass after an incubation of 72 h.
